# Aflatoxin M_1_ exposure in a fermented millet-based milk beverage ‘*brukina’* and its cancer risk characterization in Greater Accra, Ghana

**DOI:** 10.1038/s41598-022-15157-y

**Published:** 2022-07-22

**Authors:** Nii Korley Kortei, Theophilus Annan, Adjoa Agyemang Boakye, Edward Ken Essuman, Clement Okraku Tettey, Vincent Kyei-Baffour

**Affiliations:** 1 Department of Nutrition and Dietetics, School of Allied Health Sciences, University of Health and Allied Sciences, PMB 31, Ho, Ghana; 2 Food Microbiology Division, Council for Scientific and Industrial Research- Food Research Institute, P. O. Box M20, Accra, Ghana; 3 Department of Biomedical Sciences, School of Basic and Biomedical Sciences, University of Health and Allied Sciences, PMB 31, Ho, Ghana; 4 Food Chemistry and Nutrition Research Division, Council for Scientific and Industrial Research-Food Research Institute, P. O. Box M20, Accra, Ghana

**Keywords:** Biochemistry, Biotechnology, Microbiology, Natural hazards

## Abstract

*Brukina* is a millet based fermented milk product which is consumed as a beverage in Ghana. It is however prone to aflatoxin M_1_ (AFM_1_) contamination, which is a serious health challenge for low and middle-income countries in subtropical regions. This study aimed at evaluating AFM_1_ levels and cancer risks associated with *brukina* (n = 150) sampled from different locations of the Greater Accra Region of Ghana. AFM_1_ were measured with High-Performance Liquid Chromatography (HPLC) connected to a Fluorescence Detector (FLD).Cancer risk assessments were also conducted using models prescribed by the Joint FAO/WHO Expert Committee on Additives (JECFA). Out of the 150 samples analyzed for AFM_1_, 80/150 (53%) tested positive between the range 0.00 ± 0.001–3.14 ± 0.77 µg/kg. Cancer risk assessments of AFM_1_ produced outcomes which ranged between 0.64 and 1.88 ng/kg bw/day, 0.31–9.40, 0.0323, and 1.94 × 10^–3^–0.06 for cases/100,000 person/yr for Estimated Daily Intake (EDI), Hazard Index (H.I), Average Potency, and Cancer Risks respectively for all age categories investigated. It was concluded that the consumption of *brukina* posed adverse health effects on the majority of the age categories in the different locations of Greater Accra Region since the calculated H.Is were greater than one (> 1). Therefore, contamination of *brukina* with AFM_1_ should be considered a high priority in public health and Ghana’s cancer risk management actions.

## Introduction

Milk is a colloidal liquid substance that flows from the mammary glands of animals^1^. It is a vital source of both micro- and macronutrients and is naturally used to feed young mammals. Children and pregnant women are predominantly the most beneficiaries of milk^1,2^. Milk, perhaps due to its rich nutrient composition, is highly susceptible to contamination originating either from the animal, the environment, or milk handlers. It is indeed a high risk product which affords optimal conditions for the proliferation of microorganisms when stored under ambient temperature^3^. Additionally, chemical contaminants may dominate the milk through the feed or treatment of the cow or through later accidental or deliberately contamination.

*Brukina,* a type of fermented milk and millet smoothie produced and consumed in Ghana, is no exception. This beverage is produced by mixing milled and steam cooked millet with fermented cow milk and sugar^4,5^. The milk used is usually fermented spontaneously and in some cases, by back slopping with leftover fermented milk. *Brukina* gets its name from its country of origin, Burkina Faso, a neighboring country to Ghana where the product is called *dèguè*^3,6^. Boakye et al.^4^, Atinmo et al.^7^ and Baidoo and Kunadu^3^ accentuated the nutritional, medicinal, economic, and social benefits of *brukina* cannot go unnoticed as this mixture product of milk and cereal suggests a good source of essential nutrients and probiotics to support the growth and development. This is particularly important in the African region where malnutrition and hunger are very prominent thus; *brukina* represents a cost-effective meal that can provide the essential nutrients required for growth and development.

In recent times*, brukina* has gained so much popularity and patronage in Ghana and is considered one of the most successful indigenous beverages in the country, providing employment for many people especially youth. Due to its high patronage, it contends well with other common local beverages such as *nunu*, *asana*, *ice-kenkey* and *sobolo*^3^. The significant risk factors associated with contamination of *brukina* are a cause of worry. Contamination of this milk product is conjectured to originate in 2 directions; firstly, via fermented milk with AFM_1_ arising from dairy animals eating feeds contaminated by the fungal genus *Aspergillus* (*flavus* and *parasiticus*)^2,8^ and secondly via fungal contamination of the millet (cereal)^9^, ultimately resulting in possible production of AFB1, AFB2, AFG1, AFG2 and in combination with some other mycotoxins.

Aflatoxins are hazardous natural secondary metabolites produced by toxic strains of *A. flavus and A. parasiticus* (fungi of the genus *Aspergillus*)^9^ and to a lesser extent by *A. nomius*. The presence of aflatoxins in a wide range of foods is well known^9^. There are five different types; aflatoxins B_1_, B_2_, G_1_, G_2,_ and M_1_ produced primarily in cow milk by cows eating contaminated silage^10^. Aflatoxins have been reported to work concomitantly with other mycotoxins or in solitude to worsen the risk of hepatocellular carcinoma (HCC), which is reported to be the fifth most frequently occurring cancer in the world^10,11^. Epidemiological and animal studies have demonstrated that hepatitis B virus (HBV) and AFM_1_ surge the likelihood of HCC in people with hepatitis B surface antigen-positive (HBsAg+) by 3.3-fold^12,13^. Neuveut et al.^14^ asserted that pre-existing liver disease due to HBV infection compromises the ability of hepatocytes to inactivate carcinogens such as aflatoxins thus increasing the chance of HCC.

Safe limits are often set by many counties to control the quantities of aflatoxins permitted in their foods^15^. This is done presumably because of the food safety hazards that are associated with its ingestion. In addition to setting regulatory limits for mycotoxins, it is also imperative to conduct health risk assessments in the population due to dietary exposure.

Exposure assessment is defined by the Food and Agriculture Organization/World Health Organization (FAO/WHO)^16^ as a qualitative and/or quantitative assessment of the likely intake of a chemical agent through food, as well as exposure from other sources, if applicable. The methodology was used to assess scientific data in order to calculate the likelihood and severity of a negative event. Risk assessment is a widely established tool for determining the potential linkages between food chain risks and real human health risk^17^.

In Ghana, few research has been done on the prevalence of AFM_1_ in some milk products. Nonetheless, to the best of our knowledge, our work is the only one which attempts to estimate the levels and cancer risk involved with the ingestion of AFM_1_ through a millet-based fermented milk beverage (*brukina*) in Accra, Ghana. The outcomes of this paper would be expedient in advising policy makers to put their emphasis in adopting international legislations on food quality parameters and to use tools that will change the mindset of the population on risks involving fungal intoxication. The data could also provide proper health education and put emphasis on designing more effective toxigenic fungi and mycotoxin management strategies for Ghana.

## Materials and methods

### Data collection instruments

#### Sampling and sample size determination

**Figure 1:**
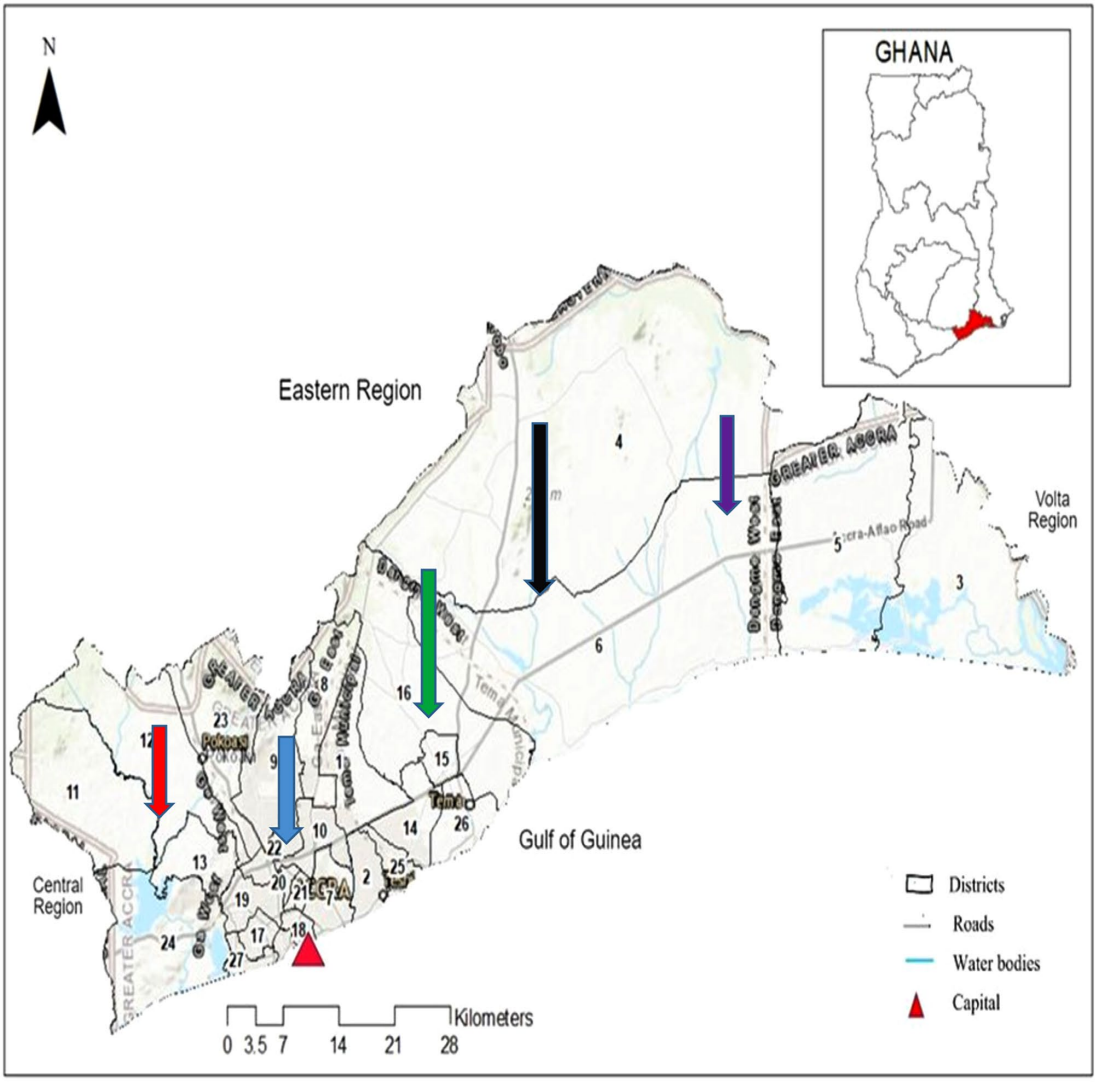
Map of Greater Accra Region and neighbouring regions (Adapted from^18^). Sampling sites are shown in red arrow (Kasoa), blue arrow (Nima), green arrow (Madina), black arrow (Ashaiman), and violet arrow (Dodowa).

The millet based fermented milk *brukina* samples were conveniently obtained from local markets in the different locations of Nima, Madina, Kasoa, Ashaiman, and Dodowa in the Greater Accra, Region of Ghana (Table 1 and Fig. 1). These areas are known to have high patronage of milk products due to nomadic inhabitants. Approximately 300 ml each of *brukina* were bought and stored in an ice chest (Thermos 7750, China) with cold packs at temperature 10 °C under aseptic conditions and transported to the laboratory in batches where they were stored in the freezer compartment of a refrigerator until these were analyzed for AFM_1_.

**Table 1:** Geographical locations and some attributes of the origin of *brukina* samples obtained from Greater Accra region of Ghana.

Region	No. of samples	Agro-ecological zones	Coordinates
Nima	30/150	Coastal Savannah	5.5820°N, 0.1984°W
Madina	30/150	Coastal Savannah	5.6731°N, 0.1664° W
Kasoa	30/150	Coastal Savannah/deciduous	5.5200°N, 2.1450°W
Ashaiman	30/150	Coastal Savannah	6.2374°N, 0.4800°W
Dodowa	30/150	Coastal Savannah/rain forest	5.8829°N, 0.0980°W

A total number of 150 *brukina* samples were used. This was determined with a Raosoft sample size calculator (http://www.raosoft.com/samplesize.html) with parameters: margin of error (8%), confidence Interval (95%), population of Accra (2,000,000) and response distribution (50%).

#### Preparation of samples

After warming at about 37 °C in a water bath, the samples were centrifuged at 2000*g* to separate fat layers and then filtered. The prepared test portion of 50 mL was transferred into a syringe barrel attached to AFM_1_ immunoaffinity column and passed at a slow steady flow rate of 1–2 mL/min. The columns were then washed with 20 mL deionized water and the air was passed through the columns to dryness. AFM_1_ was eluted with 4 mL pure acetonitrile by allowing it to be in contact with the column for not less than 60 s. The eluate was evaporated to dryness using a gentle stream of nitrogen. The residue was dissolved in 500 µL of mobile phase and filtered using a membrane filter before being injected into HPLC for quantification.

#### Chemicals and standards

The analytical standard of AFM_1_ was supplied by Sigma-Aldrich (St. Louis, MO, USA). All solvents used for the preparation of the mobile phase were HPLC grade and obtained from Merck (Darmstadt, Germany). All homogenized mixtures and eluates were filtered through Whatman no. 4 and 0.45 mm membrane filters, respectively (Whatman plc, Maidstone, UK). De-ionized water was obtained with a Millipore Elix Essential purification system (Bedford, MA, USA). EASI-EXTRACT AFM_1_ immunoaffinity columns (stored at 4 °C until use) were supplied by R-Biopharm, Rhone limited, and used for SPE and cleanup.

#### Preparation of standard solutions

A mother stock solution (0.1 μg/mL) was prepared from a standard solution of AFM_1_ (0.993 μg/mL in acetonitrile) and stored with care in the freezer. A working stock solution of 0.01 μg/mL was diluted step by step with the combined solution (acetonitrile/water, 75/25, v/v) to prepare a sequence of working solutions that were stored in vials below 4 °C for the calibration curve. Calibration solutions of 0.02 μg/kg, 0.04 μg/kg, 0.06 μg/kg, 0.08 μg/kg, and 0.10 μg/kg were used. Samples with AFM_1_ amount above the calibration range were diluted and dilution factors were applied for quantification.

### Instrumentation

Agilent high-performance liquid chromatography system (HPLC 1260 infinity series) with a quaternary pump and fluorescence detection was used for AFM_1_ quantification analysis and was carried out as per the method given by EN ISO 14501:2007^19^. Data acquisition and quantification were done using Chem station (Open Lab edition). The Agilent HPLC equipped with a fluorescence detector was set at an excitation wavelength of 360 nm and an emission wavelength of 440 nm and the column compartment (HPLC Column: TC-C18 (2), 170, 5 µm, 4.6 × 250 mm; thus, a pore size of 170, particle size of 5.0 µ, inner diameter of 4.6 mm, length of 250 mm and carbon load of 12%) temperature regulated at 35 °C. The mobile phase was a mixture of water and acetonitrile at ratios of 25:75 (v/v), respectively, and an isocratic delivery mode was employed at a flow rate of 0.8 mL min^−1^ with an injection volume of 10 µL.

### Validation

HPLC-FLD method was validated according to the guidelines of European Commission Decision 657/2002/EC for confirmatory analysis methods and the tested parameters were linearity, limit of detection (LOD), limit of quantification (LOQ), accuracy, precision, and selectivity. The linearity was assessed by constructing five-point solvent matched calibrations in triplicate for AFM_1_ standard solutions in the concentration range of 0.05 to 0.8 mg/L. Calibration curves were drawn by plotting the peak area against AFM_1_ concentration, and linearity was evaluated by linear regression analysis expressed as coefficient of determination (r^2^).

The precision of the method was estimated in terms of % RSD of three identical extractions of *brukina* samples spiked with AFM_1_ at the same as well as at three different spiking levels. Method selectivity was evaluated by analyzing AFM_1_ known negative milk matrix and reagent blank to determine any interference from endogenous substances around the retention time of the target analyte.

Method precision was estimated using intermediate precision and repeatability. For repeatability estimates, 10 different samples were taken from the same lot and each of the 10 samples spiked at the same concentration and analyzed at the same time on the same day. Three replicate measures were made for each of the 10 and the relative standard deviation (RSD) calculated.

For intermediate precision, 6 different samples were taken from the same lot and each of the 6 samples spiked at the same concentration on different days and analyzed by 3 different analysts over a 6-day period. Three replicate measures were made for each of the 6 and RSD calculated.

Mean peak area showed a proportionate increase with that of standard concentration and the calibration curves of AFM_1_ demonstrated a good regression line (r^2^ > 0.99) in the range of explored concentrations. For the recovery analysis, samples previously tested to guarantee the nonappearance of the studied mycotoxins were used in the validation procedure. The Limits of Detection for AFM_1_ ranged between 0.13 and 0.15, while Limits of Quantification ranged between 0.26 and 0.30, respectively, for both.

### Human risk assessment of exposure to AFM_1_ via consumption of *brukina* milk

#### Estimation of exposure

Estimated Daily Intake (EDI) was considered by using the mean amount of AFM_1_ derived from the milk samples, the number of samples consumed daily, and the average body weight. The EDI for mean aflatoxin was premeditated according to the following formula (1) and expressed in μg/kg of body weight/day (μg/kg bw/day)^20,21^;

1 $$\mathrm{EDI}=\frac{\text{daily} \; \text{intake} \; \left(\mathrm{food}\right) \times \text{ mean} \; \text{level} \; \text{of} \; \text{AFM}1}{\text{average} \;  \text{bodyweight}}$$

The daily intake of milk in Ghana according to Omore et al.^22^ is approximately 0.0137 kg/day (5.0 kg/year).

The different age categories according to EFSA^23^ and their corresponding estimated average weights in Ghana used in this study were done as follows; Infants—2.9 (2.5–3.2) kg^24,25^, Toddler—9.8 (7–12.6) kg^26,27^, Children—26 (24–28) kg^28,29^, Adolescents—46.25 (38.5–54) kg^30^, Adults—60.7 kg^31^.

#### Population risk characterization for aflatoxins

##### Hazard Index (H.I)

Genotoxic and carcinogenic compounds such as aflatoxins have their risk assessment fittingly computed based on the Hazard Index (H.I). The tolerance daily intake (TDI) value for AFM1 was 0.2 ng/kg/day as suggested by Kaur et al.^32^, which was calculated by multiplying the TD_50_ (threshold dose/bw) by 5000. If the H.I of AFM_1_ does not exceed 1, the consumer is presumably safe; however, if the H.I of AFM_1_ is greater than 1, the consumer may be at risk of liver cancer^33^.

2 $$\mathrm{H}.\mathrm{I}= \frac{\text{Estimated} \; \text{Daily} \; \text{Intake} \; \text{of}\; \text{AFM}1}{\text{Tolerable} \;\text{Daily}\; \text{Intake} \; \text{for} \; \text{AFM}1}$$

$$\mathrm{H}.\mathrm{I}= \frac{\mathrm{EDI }\;(\mathrm{ng}/\text{kg} \; \text{bw}/\mathrm{day})}{0.2\mathrm{ ng}/\text{kg}\; \text{bw}/\mathrm{day}}$$

The HI of AFM1 for the milk and milk products studied was estimated using the formula above.

#### Estimated liver cancer risk due to consumption of ‘brukina’ samples

The ingestion of aflatoxins can be linked to the onset of liver cancer^34,35^. Therefore, liver cancer risk estimation for Ghanaian adult consumers was calculated for aflatoxins^35,36^. This involved estimating the population cancer risk per 100,000, which is a product of the EDI value and the average hepatocellular carcinoma (HCC) potency figure from individual potencies of Hepatitis B surface antigen (HBsAg) (HBsAg-positive and HBsAg-negative groups).

The JECFA^37^ estimated potency values for AFM_1_ which corresponded to 0.3 cancers/year/ 100,000 population ng/kg bw/day (uncertainty range: 0.05–0.5) in HBsAg-positive individuals and 0.01 cancers/year/100,000 population ng/kg bw/day (uncertainty range: 0.002–0.03) in HBsAg-negative individuals^35^ were adopted for this calculation. Moreover, the average HBsAg+ prevalence rate of 7.74% (adult—8.36%, 14.3%—adolescents, 0.55%—children) for Ghana^38,39^ was adopted and 92.26% (100–7.74%) was extrapolated for HBsAg-negative groups. Hence, the average potency for cancer in Ghana was estimated as follows according to Eq. (7) as prescribed by^35^ and^36^:

3 $$\begin{aligned}   Average\;Potency & = \left[ {0.03 \times HBsAg {\text{-}} negative\;individuals\;in\;Ghana} \right] + \left[ {0.01 \times HBsAg {\text{-}} positive\;individuals/prevalance\;rate\;in\;Ghana} \right] \hfill \\    & = \left( {0.3 \times 0.077} \right) + \left( {0.01 \times 0.9226} \right) \hfill \\    & =  0.0323 \hfill \\  \end{aligned}$$

Thus, cancer risk (cancers per year per 100,000 population per ng aflatoxin /kg bw/day) was estimated using the following formula in Eq. (4)^34,36^:

Thus, the population risk was estimated using the following formula in Eq. (4):

4 $$\text{Cancer} \; \text{Risk}=\text{Exposure} \; (\text{EDI}) \times \text{Average} \; \text{potency}$$

### Statistical analysis

The aflatoxin concentrations were calculated using regression analysis from the curves generated from the standards of aflatoxin M_1_ with Excel for Microsoft Windows (version 10). One sample *t*-test was used to compare the means obtained at a 95% confidence interval and 5% level of significance. The statistical results were summarized as median, standard deviation, variance, skewness, standard error of skewness, kurtosis and standard error of kurtosis and mean values (range from the 25th percentile to the 75th percentile). SPSS 22 (Chicago, USA) was used in the analysis of data. Deterministic risk assessment model calculations for aflatoxins, dietary exposure (Estimated Dietary Intake), H.I values, Average potency, and cancer risk were calculated.

## Results

The mean recovery percentage of AFM1 in spiked milk samples were found between 80.5 and 84.07% with % RSD from 3.19 to 5.42. Since, the recoveries and % RSD were within the EC regulation.

The summary of statistics of the number of food samples contaminated with AFM_1_ is presented in Table 2. The level of occurrence of the AFM_1_ ranged between 0–2.30 µg/kg, 0–3.02 µg/kg, 0–3.14 µg/kg, 0–2.11 µg/kg, and 0–2.14 µg/kg respectively for Nima, Kasoa, Madina, Ashaiman and Dodowa in the Greater Accra Region.

**Table 2:** Summary of statistics of AFM_1_ in fermented milk beverage *brukina* obtained from five different areas of Greater Accra region, Ghana.

	Nima	Kasoa	Madina	Ashaiman	Dodowa
No. of samples	30	30	30	30	30
Mean	0.2737	0.7973	0.5017	0.6263	0.5780
Std. error of mean	0.098	0.16071	0.140	0.12131	0.12318
Median	0.0400	0.6200	0.000	0.5850	0.0000
Std. deviation	0.54218	0.88022	0.767	0.6644	0.6746
Variance	0.294	0.775	0.589	0.441	0.455
Skewness	2.493	0.821	1.834	0.615	0.612
Std. error of skewness	0.427	0.427	0.427	0.427	0.427
Kurtosis	6.265	− 0.360	3.762	− 0.828	− 0.957
Std. error of kurtosis	0.833	0.833	0.833	0.833	0.833
Range	2.30	3.02	3.14	2.11	2.14
**Percentiles**					
25	0.000	0.000	0.000	0.000	0.000
50	0.040	0.6200	0.000	0.5850	0.000
75	0.160	1.4750	0.930	1.1125	1.1275

Nima recorded comparatively less mean and median values of AFM_1_ concentrations than all localities (Kasoa, Madina, Ashaiman, and Dodowa) investigated. The skewness and kurtosis were 2.493 and 6.265, respectively and showed that the data set of AFM_1_ obtained in this town was asymmetrical and heavy-tailed (Table 2). The lower and upper limits were 0.0712 and 0.4761, respectively, and showed significant differences (p < 0.05) (Table 3).

**Table 3:** Statistics of the one-sample *t*-test of *brukina* samples from different parts of the Greater Accra region, Ghana.

	Positive AFM_1_ (%)	*t*	df	Sig. (2-tailed)	Mean difference	95% confidence interval of the difference	
						Lower	Upper
Nima	17/30(56.7)	2.765	29	0.010	0.27367	0.0712	0.4761
Madina	12/30(40)	3.581	29	0.001	0.50167	0.2152	0.7882
Kasoa	19/30(63.3)	4.961	29	0.000	0.79733	0.4687	1.1260
Ashaiman	18/30(60)	5.163	29	0.000	0.62633	0.3782	0.8744
Dodowa	14/30(46.7)	4.692	29	0.000	0.57800	0.3261	0.8299

For Kasoa, greater values of mean and median AFM_1_ concentrations than Madina, Ashaiman, and Dodowa were recorded from the summary statistics. Values of 0.821 and − 0.360 were recorded as skewness and kurtosis and implied moderate skewness and light-tailed. The upper and lower limits were 0.4687 and 1.1260. Values significantly differed (p < 0.05) (Tables 2 and 3).

The mean and median concentrations of AFM_1_ recorded in Madina were comparatively greater than Nima but lesser than Kasoa, Ashaiman, and Dodowa. While the data set showed symmetrical and light-tailed as, the skewness and kurtosis were 1.834 and 3.762, respectively (Table 2). Values of 0.2152 and 0.7882 were recorded as upper and lower limits. There were significant differences (p < 0.05) observed (Table 3).

For Ashaiman, we recorded greater mean and median concentrations of AFM_1_ than Dodowa, Madina, and Nima. However, the values were lesser than Kasoa, data set for Ashaiman was fairly symmetrical and light-tailed; 0.615 and − 0.67 for skewness and kurtosis, respectively. Upper and lower limits of 0.3782 and 0.8744 were, respectively, recorded. There were significant differences (p < 0.05) (Tables 2 and 3).

Lastly, for Dodowa, we recorded lesser mean and median values of the concentrations of AFM_1_ than Kasoa and Ashaiman but not Nima and Madina. The data set for Dodowa was fairly symmetrical and light-tailed; 0.612 and − 0.597 for skewness and kurtosis, respectively. Upper and lower limits of 0.3261 and 0.8299 were, respectively, recorded. There were significant differences (p < 0.05) (Tables 2 and 3).

Regarding the frequency and (percentage %) of positive AFM_1_ in contaminated *brukina* samples, values recorded for overall positive samples was 80/150 (53%) while the different locations recorded values of 17/30 (56.7%), 12/30 (40%), 19/30 (63.3%), 18/30 (60%) and 14/30 (46.7) for Nima, Madina, Kasoa, Ashaiman and Dodowa respectively (Table 3).

### Risk assessment

The Estimated Daily Intakes (EDI) of AFM_1_ in the *brukina* samples from Nima were 0.64, 0.38, 0.14, 0.079, and 0.061 ng/kg bw/day for infants, toddlers, children, adolescents, and adults respectively. The Harzard Index (H.I) values recorded were 3.20, 1.90, 0.70, 0.40, and 0.31, respectively, and implied an adverse health risk for infants and toddlers. The average potency of the aflatoxins was 0.0323 aflatoxins ng/kg bw/day and produced cancer risks of 0.0206, 0.0122, 4.52 × 10^–3^, 2.55 × 10^–3^, and 1.97 × 10^–3^ cases/100,000 person/yr respectively (Table 4).

**Table 4:** Risk evaluation for AFM_1_ via consumption of *brukina* samples obtained from Greater Accra region of Ghana.

Locality		Av. body wgt. (kg)	Estimated daily intake (EDI) (ng/kg bw/day)	Hazard Index (H.I)	Cancer risk (cases/100,000 person/yr)
Nima	Infants (0–11 mths)	2.9	0.64	3.2	0.0206
	Toddlers (12–35 mths)	9.8	0.38	1.90	0.0122
	Children (36 mths–10 yrs)	26	0.14	0.70	4.52 × 10^–3^
	Adolescents (11–17 yrs)	46.25	0.079	0.40	2.55 × 10^–3^
	Adults (18–64 yrs)	60.7	0.061	0.31	1.97 × 10^–3^
Madina	Infants (0–11 mths)	2.9	1.185	5.93	0.038
	Toddler (12–35 mths)	9.8	0.70	3.50	0.02261
	Children (36 mths–11 yrs)	26	0.264	1.30	8.52 × 10^–3^
	Adolescents (11–17 yrs)	46.25	0.149	0.75	4.813 × 10^–3^
	Adults (18–64 yrs)	60.7	0.113	0.57	3.65 × 10^–3^

Mean of AFM_1_—Nima = 0.27 µg/kg, Madina = 0.5017 µg/kg.

Daily intake of milk for infants was halved (0.5 × 0.0137 kg).

Average body weights were obtained from the different ranges referenced by the authors.

Average potency for AFM_1_ = 0.0323.

1 µg = 1000 ng.

*H.I* Hazard Index.

Madina recorded EDI values of 1.185, 0.70, 0.264, 0.149, and 0.113 ng/kg bw/day for infants, toddlers, children, adolescents, and adults respectively. H.I values were 5.93, 3.50, 1.30, 0.75, and 0.57 and pointed at an adverse health risk for infants, toddlers, and children. The average potency was the same. Cancer risks of 0.038, 0.02261, 8.52 × 10^–3^, 4.813 × 10^–3^, and 3.65 × 10^–3^ cases/100,000 person/yr respectively for these age categories were recorded (Table 4).

For Kasoa, the EDI values recorded for infants, toddlers, children, adolescents, and adults were 1.88, 1.1142, 0.420, 0.236, and 0.180 ng/kg bw/day respectively. H.I values recorded were 9.40, 5.57, 2.10, 1.18, and 0.90, respectively, which showed adverse health risk for infants, toddlers, children, and adolescents. The average potency was the same as other regions, while the cancer risks were 0.07, 0.04, 0.014, 8.08 × 10^–3^, and 6.14 × 10^–3^ cases/100,000 person/yr respectively (Table 5).

**Table 5:** Risk evaluation for AFM_1_ via consumption of *brukina* samples obtained from Greater Accra region of Ghana.

Locality		Av. body wgt. (kg)	Estimated daily intake (EDI) (ng/kg bw/day)	Hazard Index (H.I)	Cancer risk (cases/100,000 person/yr)
Kasoa	Infants (0–11 mths)	2.9	1.88	9.40	0.0607
	Toddlers (12–35 mths)	9.8	1.1142	5.57	0.0359
	Children (36 mths–10 yrs)	26	0.420	2.10	0.01356
	Adolescents (11–17 yrs)	46.3	0.236	1.18	7.62 × 10^–3^
	Adults (18–64 yrs)	60.7	0.180	0.90	5.814 × 10^–3^
Ashaiman	Infants (0–11 mths)	2.9	1.479	7.40	0.05
	Toddlers (12–35 mths)	9.8	0.875	4.38	0.03
	Children (36 mths–10 yrs)	26	0.330	1.65	0.01
	Adolescents (11–17 yrs)	46.3	0.185	0.93	6.46 × 10^–3^
	Adults (18–64 yrs)	60.7	0.141	0.71	4.85 × 10^–3^
Dodowa	Infants (0–11 mths)	2.9	1.365	6.83	0.044
	Toddlers (12–35 mths)	9.8	0.808	4.04	0.0260
	Children (36 mths–10 yrs)	26	0.305	1.53	9.85 × 10^–3^
	Adolescents (11–17 yrs)	46.3	0.171	0.86	5.523 × 10^–3^
	Adults (18–64 yrs)	60.7	0.130	0.65	4.199 × 10^–3^

Means of aflatoxins M1—Kasoa—0.79 µg/kg, Ashaiman—0.62 µg/kg, Dodowa—0.5780 µg/kg.

Daily intake of milk for infants was halved (0.5 × 0.0137 kg).

Average Body weights were obtained from the different ranges referenced by the authors.

Average potency for AFM_1_ = 0.0323.

1 µg = 1000 ng.

*H.I* Hazard Index.

At Ashaiman, the EDI values recorded for infants, toddlers, children, adolescents, and adults were 1.479, 0.875, 0.330, 0.185, and 0.141 ng/kg bw/day respectively. H.I values recorded were 7.40, 4.38, 1.65, 0.93, and 0.71, respectively, implying an adverse health risk for infants, toddlers, and children. The average potency was the same as other regions, while the cancer risks were 0.05, 0.03, 0.01, 6.46 × 10^–3^, and 4.85 × 10^–3^ cases/100,000 person/yr respectively (Table 5).

Lastly, for Dodowa, the EDI values recorded for infants, toddlers, children, adolescents, and adults were 1.88, 1.114, 0.42, 0.236, and 0.180 ng/kg bw/day respectively. H.I values recorded were 9.40, 5.57, 2.10, 1.18, and 0.90, respectively, and suggested an adverse health risk for infants, toddlers, children, and adolescents. The average potency was the same as other regions, while the cancer risks were 0.044, 0.0260, 9.85 × 10–3, 5.523 × 10^–3^, and 4.199 × 10^–3^ cases/100,000 person/yr respectively (Table 5).

## Discussion

According to Bhaskar^40^, all around the globe, consumption of unsafe food results in approximately 420,000 deaths annually and is the cause of more than 200 diseases ranging from diarrhoea to cancer. *Brukina* is consumed by most Ghanaians as a beverage served chilled with ice blocks and is conjectured to be prepared under unhygienic conditions. Again, the milk used for its preparation is repeatedly prone to contamination with AFM_1_ making it unsafe_._ In these present investigations, Nima had a comparatively low mean concentration of 0.27 µg/kg and significantly differed (p < 0.05) from the mean concentrations of the other towns which had greater (> 0.5) concentrations. Conversely, there was an observed a greater number of positive samples got from Nima, Kasoa as well as Ashaiman. Our results point at comparatively moderate levels of AFM_1_ contamination in *brukina* samples from different locations of Greater Accra region of Ghana. They were within the range of 0–3.14 µg/kg (3140 ng/kg) and compared satisfactorily well with some published findings of some researchers around the globe.

From Ghana, Addo-Boadu^41^ recorded AFM_1_ levels in a range of 0.35–3.76 µg/L (350–3760 ng/L) in raw milk and milk products samples from the Greater Accra Region. In Tanzania, studies conducted on raw cow milk samples revealed 83.3% (31/37) of aflatoxin contamination in cows that fed on sunflower cake in the range of 0.026–2.007 µg/kg (26–2007 ng/kg) (exceeding both Tanzania’s and EC allowable value which is 0.05 µg/kg^42^.

Several studies have reported the occurrence of AFM_1_ in milk and dairy products. Milk samples from urban centers in Kenya contained AFM_1_ up to 6800 ng/L^43^. In Sudan, 95% of milk was contaminated with AFM_1_ ranging between 220 and 6800 ng/L^44^, whereas 6–527 ng/L of AFM_1_ was detected in 15% of cow milk samples from Cameroon^45^. The concentration of AFM_1_ varied between 150 and 170 ng/L in commercial and rural milk in South Africa^46^, while 100% of milk samples in Nigeria contained AFM_1_ and the levels were within the range of 0.004–0.845 µg/L (4–8450 ng/l). Goncalves et al.^47^ reported AFM_1_ levels in fresh bovine milk to be in between the range of 0.09–3.385 µg/L (90–3385 ng/L) per their work. The least amount of data is available from African countries, nonetheless the available data suggest the highest prevalence and frequent detection levels^48^. Lower values were recorded by^49^ in milk from Sudan and found 33% of the milk samples with the highest occurrence (82.4%) in cow milk (35.3% ranged between 0.05 and 0.1 μ/kg and 47.1% ranged between 0.1 and 0.15 μ/kg) and milk samples from a camel in semi-intensive systems (15.6% ranged between 0.05 and 0.1 μg/kg). Again, all samples of milk from traditional nomadic systems indicated an absence of AFM_1_.

Makun et al.^50^ also reported contamination of raw cow milk with AFM_1_ at levels higher than the EU permitted levels in Nigeria. In their study, contamination of raw cow milk (from a nomadic cow) with AFM_1_ ranged from 0.0109 to 1.3543 µg/L (10.9–1354.3 ng/L) with and an average concentration of 0.5308 ± 0.0938 µg/L (530.8 ng/L). For commercial cow’s milk, AFM_1_ contamination ranged between 0.0464–0.0992 (46.4–99.2 ng/L) and 0.0584 ± 0.0052 µg/L (58 ng/L) as the mean. A similar trend regarding levels of AFM_1_ contamination of 0.05 µg/kg has also been reported in a study conducted in Brazil by^51^. From Egypt, Amer et al.^52^ reported a prevalence of 38 positive samples with a range of 0.023–0.073 µg/L (23–73 ng/L) in raw milk. Also, Rahimi et al.^53^ reported mean values of 0.0601, 0.0319, 0.0190, 0.0281, and 0.0301 µg/L (60.1, 31.9, 19.0, 28.1, and 30.1 ng/L) respectively for raw cow, water buffalo, camel, sheep, and goat milk from Iran.

Milk in Europe is time and again analyzed for AFM_1_ and is also averred to be the safest^54^. From Portugal, Duarte et al.^55^ reported values of range n.d–0.069 µg/L (nd–69.0 ng/L) in 99.4% positive raw milk samples. From Spain, Rodriguez-Blanco et al.^56^ and Cano-Sancho et al.^57^ reported ranges of n.d–0.2 µg/L (n.d–200 ng/L) and 0.009–1.36 µg/L (9–1360 ng/L) respectively for raw milk samples. From Serbia, Kos et al.^58^ and Tomasevic et al.^59^ reported ranges of 0.01–1.2 µg/L (10–1200 ng/L) and 0.09–0.145 µg/L (90–1450 ng/L) respectively for milk. Furthermore, in Croatian milk, values of 0.006–0.027 µg/L (6–27 ng/L) were reported by^60^. AFM_1_ levels were recorded in Italy by^61^ and^62^, all pointed at results below 0.05 µg/kg. Available data shows an irregular pattern of results which does not portray low levels to suggest total safety as no amount of AFM_1_ is Generally Regarded as Safe (GRAS).

In other parts of the world, Iha et al.^63^ from Brazil, reported 83% of the milk samples tested positive for AFM_1_, in a range of 0.008 to 0.760 ng/g and in India, almost half of the analyzed milk was contaminated, with 44% being above EU limit^64^. Greater quantities of AFM_1_ have been reported across the globe. Lee et al.^65^ from South Korea reported values of 0.22–6.9 µg/l (220–6900 ng/L) in raw milk. From Pakistan, Iqbal et al.^66^ reported values of 0.02–3.09 µg/l (20–3090 ng/L).

The contamination rate and levels of AFM_1_ in fermented milk obtained in this study may be because dairy animals kept in local dairy farms were fed with compound rations stored under poor conditions and may have favored the growth of toxicogenic fungi expressly *Aspergillus sp*. which can in due course, be contaminated with aflatoxins. Again, hot and humid climatic conditions are very conducive for fungal invasion, growth, and production of mycotoxins including aflatoxins in food and feed commodities^67^. Unseasonal rains and related flash floods are widespread, and this increases the moisture content of the grains and other feedstuff, and therefore its vulnerability to fungal attacks. Indeed, several previous reports indicated the presence of high levels of aflatoxins in dairy animals' feed and ingredients from Ghana.

Moreover, most of the dairy farmers prefer to feed cereals (maize, wheat, etc.) or agricultural or oilseed byproducts (peanuts, soybean, etc.) to their dairy animals, and such aflatoxin susceptible feed materials constitute more than 70% of cattle feed^67^. Therefore, if such high aflatoxin contaminated feedstuff is included in the diet of dairy animal's, there is always a great possibility of AFM_1_ appearing in milk at high levels. Other probable factors which may play an important role in the high levels of AFM_1_ in milk in this study include poor farm management practices especially feed storage practices, no legal limits of aflatoxins exist for livestock feed, and lack of knowledge among dairy farmers concerning aflatoxins.

Aflatoxin exposure early in life has been associated with impaired growth, particularly stunting^68^. Furthermore, early exposure to aflatoxins is a potential risk for synergistic interactions with other toxins as subjects grow^69,70^. Weaning is a transition period of a child from breast milk to other sources of food, which often results in a marked decrease in nutrient intake in developing countries^71^. One possible variable contributing to poor child health in developing countries is the increased exposure to aflatoxin-contaminated foods following weaning^72^.

Comparatively greater quantities of AFM_1_ were detected in other parts of the world by other researchers. In the global context, AFM_1_ levels found in Ghanaian milk are moderate. Flores-Flores et al.^48^ have reviewed the presence of AFM_1_ in cow's milk from various parts of the world. Of the 22,189 milk samples analyzed that were taken into account, at least 9.8% of them (2190 samples) exceeded the maximum AFM_1_ content established by the EU. Regarding the number of noncompliant samples per continent, 1709 came from Asia, 253 from Africa, 119 from Europe, and 109 from America. Gizachew et al.^73^ and Skrbic et al.^74^ emphasized several factors such as geographical region, season, type and quality of feed, feed storage conditions, and processing methods and conditions that are responsible for the variability of AFM_1_ in milk and dairy products. Lack of fresh forage as feed might have led to longer storage of hay or feed leading to contamination of *Aspergillus s*p. leading to AFB_1_ contamination.

### Risk assessment

The public health significance of AFM_1_ levels in milk has never been fully revealed. The risk of cancer development involved with the prolonged ingestion of mycotoxin, which is by and large linked to its concentration. In the present study, the age categories of infants, toddlers, children, and adolescents were found to be the most at risk of adverse health effects (Hepatocellular carcinoma) while the adult populations were not at risk. Whereas some research works have found an association between stunting and aflatoxins^75^, proof of its causes is still absent. In Kenya, an association between AFM_1_ exposure and lower height-forage scores. Similarly, a study in Iran showed that infants of mothers which had AFM_1_ in their breast milk had lower height-for-age scores^76^. A recent scoping review by Soriano et al.^77^ showed the presence of these aflatoxins appeared in greater proportion in kwashiorkor in children and in different organs and biological samples including brain^78^, heart^79^, kidney^80^, liver^79^, lung^81^, serum^82^, stool^83^ and urine^45,82^ whereas in the marasmic-kwashiorkor they were detected in similar parts.

Aflatoxins are unaffected by many food processing techniques such as boiling or pasteurization, etc. as they are heat stable^84^. There is always a risk involved with their association with food or feed. Risk estimations as explained by Liu and Wu^85^ as well as Kuiper-Goodman^86^ are modeled to predict the magnitude of adverse health implications of mycotoxin exposure and guide food regulators to set thresholds for these toxins in foodstuffs. H.I results obtained in this study implied a high risk for infants, toddlers, children, and adolescents (total aflatoxins).

Considering the EDI values obtained in a study by Addo-Boadu^41^ in Ghana for infants i.e., 3.679 ± 2.213 and 2.445 ± 2.001 ng/kg bw/day, it exceeded 1 ng/kg bw/day by far and indicated the serious risk of AFM_1_ through raw cow milk consumption for this age category.

Our findings corroborated published findings of Kaur et al.^32^, which indicated EDI and HCC values of 2.30 and a range of 0.0020–0.0106, respectively. Their health risk assessment revealed that customers in the research area, particularly youngsters, are at a higher risk of AFM1 infection due to their low body weight and increased milk consumption.

Recently from Malawi, Njombwa et al.^13^ reported a probable mean daily exposure to AFM_1_ for adults as 4.98 ± 7.25 ng/kg bw/day and almost double for children (8.28 ± 11.82 ng/kg bw/day). The estimated risk of AFM_1_-induced HCC associated with consumption of milk among children and adults were 0.038 and 0.023 cases per 100,000 individuals per year, respectively. Their results suggested a low risk of hepatocellular carcinoma (HCC).

The incidence of liver cancer in Iran was 3.53 cancers per year per 10^5^ persons or 3530 cancers/yr/10^8^ persons^87^ and AFM_1_ intake through yogurt contributed 0.023–0.048 cancers/yr/10^8^ person for mean consumers and 0.028–0.069 cancers/yr/10^8^ person for high consumers. Therefore, their findings indicated AFM_1_ in yogurt contributed a slight part to the overall incidence of liver cancer in the Iranian population. The intake of AFM_1_ and liver cancer incidence due to the consumption of this mycotoxin through yogurt and milk have been reported in other countries including China, Spain, Greece, and Serbia^57,88,89^.

The range of liver cancer incidence or hepatocellular carcinoma (HCC) due to AFM_1_ intake through milk and yogurt was 0.025–0.033 case or cancers/yr/10^8^ person in China, was similar to the results of this study in Serbia and Greece was 3.6–0.4.7 and 0.7–0.9 case or cancers/yr/10^8^ person, respectively that it was higher than the current study. These distributes were related to the AFM_1_ level and consumption value of yogurt.

Studies by Serraino et al.^90^ from Italy, the EDI of AFM_1_ in different population groups were in the range of 0.025–0.328 ng/kg bw/day, based on the average consumption levels and weighted mean contamination of milk in the study period. The estimated fractions of HCC incidences attributable to AFM_1_ intake were 0.005 and 0.004 cases per 100,000 individuals in the 0–0.9 and 1–2.9-year age groups, respectively, and below 0.004 cases in the other age categories which posed adverse health consequences.

Trevisani et al.^91^ in a related study, reported 0.011–0.057 cases/100,000 people in different age categories in an Italian population. The estimated fraction of the incidence of HCC in the Italian population projected a slight increase in cases due to milk consumption.

The Survey of the AFM_1_ contamination level of commercially available pasteurized milk and raw milk in Japan showed that the average concentration ± standard deviation of AFM_1_ was 0.009 ± 0.0004 μg/kg in commercially available milk and 0.0074 ± 0.0047 μg/kg in raw milk. The survey of the AFM_1_ contamination level of powdered infant formula indicated that the average The concentration of AFM_1_ was 0.002 μg/kg when converted to the concentration in the formula.

Estimation of carcinogenic risk based on the lifetime exposure to AFM_1_ calculated from these values suggest that the risk is extremely low in the present situation^92^.

It is worthy to note that despite the higher AFM_1_ levels found in milk of African origin, presumably due to the ability of *Aspergillus* species to flourish better under tropical climate^93,94^ thus producing the parent compound AFB_1_, which is metabolized into AFM_1_ by mammals and subsequently secreted into milk^95^ viz-a-vis industrialized countries in temperate regions, it appears that the dietary exposure could generally be low due to low amount of milk consumed. For instance, JECFA^96,97^ estimated milk consumption per person at 42 mL/day for African countries, which was about 8–9 times lower compared to a consumer in the industrialized countries. Thus, at 60 kg body weight, average daily exposures were lower in African countries (0.002 ng/kg bw/day) compared to 0.11 ng/kg bw/day for consumers in developed countries^96,97^. For the reason that, to a large extent aflatoxins possess carcinogenic potential, JECFA^98^ established that daily exposure, not exceeding 1 ng/kg bw, contributes to the risk of liver cancer. In the face of the anticipated risk of cancer incidence that can be gotten from AFM_1_ in this study, the effects of AFM_1_ on health, and especially the combined effects of mixtures of mycotoxins, the additive effects of aflatoxins, other dietary contaminants, alcohol consumption, and poor diet on cancer risk remains largely unknown.

The range of results for AFM1 in *brukina* obtained from Greater Accra region may vary from that of different regions since conditions of silage storage and feed that influence the growth and survival of the *Aspergillus* species, may change and therefore change the contamination levels.

## Conclusion

From the findings of this study, it can be deduced that a moderate percentage 53% of millet- based fermented milk beverage *brukina* samples collected in different locations of Greater Accra Region of Ghana proved to have AFM_1_ contents, it further showed a public health concern considering the adverse health especially hepatocellular carcinoma (HCC) outcome of the health risk assessments since the calculated H.Is were greater than one (> 1) in mostly infants (all localities), toddlers (all localities), children (Madina, Kasoa, Ashaiman, Dodowa) and adolescents (Kasoa, Dodowa) age categories.

In spite of the important role of milk, especially dairy products in the human diet, there is a great concern about the presence of AFM_1_ in milk and dairy products. Additional negative health effects of AFM_1_ justify its continuous monitoring and update of risk assessment. Hence, it is imperative to use fast methods in the detection of AFM_1_ in *brukina* as well as milk and dairy products. Ghanaian public health authorities have to monitor ceaselessly to detect AFM_1_ contamination and need to be suppressed to an ALARA (as low as reasonably achievable) level.

Although the sampling sites chosen in the present study were representative enough of Accra to draw sufficient statistical conclusions, many more sites could have been added. Again, *brukina* samples cannot be obtained in all areas but only in particular areas where cattle are reared and so makes it difficult to access.

Some novel risk assessment approaches like simulated distribution, Log-N, and some others could be employed or adapted as possible tools or areas for future studies.

## Data Availability

Data sharing is not applicable to this article as no datasets were generated or analyzed during the current study.

## Abbreviations

AFM_1_: Aflatoxin M_1_
HPLC-FLD: High perfomance liquid chromatographer-flourescence detector
WHO: World Health Organization
FAO: Food and Agriculture Organization
JECFA: Joint Expert Committee on Food Additives
SPSS: Statistical package for social sciences
EDI: Estimated daily intake
TDI: Tolerable daily intake
HCC: Hepatocellular carcinoma
LOD: Limit of detection
LOQ: Limit of quantification
HBsAg+: Hepatitis B surface antigen positive
HBsAg−: Hepatitis B surface antigen negative
ALARA: As low as reasonably allowed
